# Risk Factors, Co-Morbidities and Treatment of In-Hospital Patients with Atrial Fibrillation in Bulgaria

**DOI:** 10.3390/medicina54030034

**Published:** 2018-05-25

**Authors:** Stefan Naydenov, Nikolay Runev, Emil Manov, Daniela Vasileva, Yavor Rangelov, Nadya Naydenova

**Affiliations:** 1 Department of Internal Diseases “Prof. St. Kirkovich”, Medical University of Sofia, Sofia 1431, Bulgaria; nrunev@abv.bg (N.R.); doctor_emil_manov@abv.bg (E.M.); daniela.g.vasileva@gmail.com (D.V.); yavor.dok@abv.bg (Y.R.); 2 Clinic of Cardiology, UMHAT “St. Ekaterina”, Medical University of Sofia, Sofia 1431, Bulgaria; nadma@abv.bg

**Keywords:** atrial, fibrillation, in-hospital, embolic, risk, prevention

## Abstract

Background and objectives: Atrial fibrillation (AF) is the most common arrhythmia worldwide and a major risk factor for cardiovascular complications. Our study aimed to investigate the prevalence, risk factors, demographics, co-morbidities and treatment of AF among in-hospital Bulgarian patients. Materials and Methods: A cross-sectional study including 1027 consecutive patients (*n* = 516, 50.2% males) with a mean age of 67.6 ± 11.3 years, hospitalized for any reason from 1 May until 31 December 2016 in one of the largest internal clinics in Bulgaria, was carried out. Results: Atrial fibrillation was diagnosed in 634 (61.7%) patients. The prevalence of modifiable AF risk factors was as follows: heart failure, 98.9%; arterial hypertension (HTN), 93.5%; valvular heart disease, 40.9%; chronic lung disease, 26.7%; type 2 diabetes mellitus, 24.9%; thyroid disease, 16.9%; and ischemic heart disease, 11.2%. Univariate logistic regression analysis identified the following risk factors with strongest impact on AF: left ventricular ejection fraction <40% (odds ratio (OR) = 1.951, 95% confidence interval (CI) 1.208–3.151), valvular heart disease (OR = 1.926, 95% CI 1.134–3.862), left ventricular ejection fraction 40–49% (OR = 1.743, 95% CI 1.248–3.017), HTN (OR = 1.653, 95% CI 1.092–3.458). History of ischemic stroke was present in 14.4% of the patients with AF. Oral antithrombotic drugs were prescribed to 85.7%: direct oral anticoagulants to 37.9%, vitamin K antagonists to 43.2%, and antiplatelets to 4.6%. Heart rate control medications and antiarrhythmics were prescribed to 75.4% and 40.2%, respectively. Conclusions: Atrial fibrillation was highly prevalent among our study population. Reduced and mid-range left ventricular ejection fraction, valvular heart disease, and HTN were the risk factors with the strongest association with AF. Although a large number of our AF patients were administered antithrombotic treatment, the prescription rate of oral anticoagulants should be further improved.

## 1. Introduction

Atrial fibrillation (AF) is the most common rhythm disorder among the general population [1,2,3,4]. The estimated number of AF patients worldwide is ~33.5 million, with higher incidence and prevalence in the developed countries [1,2,3,4]. The number of patients with this arrhythmia is expected to reach ~17 million in Europe by 2030, with >200,000 newly diagnosed patients annually [5,6,7].

Despite the better risk stratification and the new therapeutic strategies, a large number of AF patients continue to be at high risk for cardiovascular complications [1,3,8]. All-cause mortality is increased twice in females and 1.5-fold in males with AF compared to individuals in sinus rhythm after statistical adjustment for other risk factors [1,7]. While appropriate oral anticoagulation could decrease substantially the death rate due to ischemic stroke, other cardiovascular complications/events such as heart failure (HF) and sudden cardiac death are not sufficiently influenced even by the current therapeutic guidelines [1,8,9].

The huge medical, social and economic impact of AF gave us grounds to conduct an observational cross-sectional study among in-hospital patients, hospitalized for any reason in one of the largest internal clinics in Bulgaria, aiming to investigate: (1) The prevalence of AF; (2) Risk factors (RF) for occurrence of this arrhythmia, demographics and co-morbidities in AF patients; (3) Antiarrhythmic and rate control treatment; (4) Antithrombotic stroke prevention. Another reason for us to conduct this study was the lack of sufficient and reliable data about patients with AF from large national registries, clinical and/or epidemiological studies in Bulgaria.

## 2. Experimental Section

An observational cross-sectional study, including 1027 consecutive patients, of them 516 (50.2%) males, hospitalized for the period 1 May to 31 December 2016. The mean age of the study population was 67.6 ± 11.3 (24–92) years. The study was approved by the local ethics committee and conducted in accordance with the ethical standards, laid down in the 1964 Declaration of Helsinki and its later amendments, guidelines for good clinical practices, and local regulations (ethical approval number 124/09.03.2016). All participants signed an informed consent before enrollment in our study.

### 2.1. Patients’ Selection

Patients meeting the following eligibility criteria were considered for enrolment in the study.

Inclusion criteria: All consenting patients hospitalized in our clinic for any reason except acute cerebrovascular accidents for the recruitment period.

Exclusion criteria: 1. Mentally disabled patients (irrespective of the reason) unable to understand or sign the written informed consent. 2. Patients unwilling to sign an informed consent for participation in our study for any reason.

Patients’ information was collected in a structured questionnaire form including demographic characteristics, socio-economic data, medical history about complaints, RF, comorbidities and medical treatment. We gathered the necessary data directly from the patient’s medical history and/or from the available medical documentation. The instrumental investigations included electrocardiography (ECG), transthoracic echocardiography, X-ray and the following laboratory parameters (fasting): full blood count with differential, K^+^, Na^+^, blood glucose, creatinine, uric acid, creatine kinase, troponin T, alanine and aspartate aminotransferase, total cholesterol, low-density and high-density cholesterol, triglycerides, prothrombin time activity, international normalization ratio, total protein and albumin, free thyroxine, thyroid-stimulating hormone. If patient’s symptoms suggested HF, but the results from the physical examination, X-ray and echocardiography were inconclusive about presence of this syndrome, we examined also N-terminal pro b-type natriuretic peptide (NT-proBNP).

In patients with symptoms, signs and X-ray data for HF (+NT-proBNP examined in some of them) we applied the following echocardiographic criteria to establish the diagnosis “Heart failure with preserved ejection fraction” (HFpEF):

1. Left-ventricular ejection fraction ≥50%.
2. Presence of ≥3 of the following criteria:

- Septal e’ velocity ≤7 cm/s or lateral e′ velocity ≤10 cm/s;
- Average E/e′ ≥14;
- Tricuspid regurgitation velocity ≥2.8 m/s;
- Left atrial volume index ≥34 mL/m^2^;
- Left ventricular mass index ≥115 g/m² for males and ≥95 g/m² for females;
- E acceleration >1900 cm/s;
- Isovolumetric relaxation time ≤65 ms;
- Deceleration time of the pulmonary venous diastolic velocity ≤220 ms.

All patients in sinus rhythm at admission and without previous history and/or medical documentation for AF episodes, were examined by 24-h Holter-ECG monitoring within 48 h of their hospitalization. Additional instrumental investigations were conducted when necessary (individual decision of the treating physician).

### 2.2. Statistical Analysis

The sample size of our study was defined by OpenEpi-free sample size calculator, available at: http://web1.sph.emory.edu/cdckms/sample%20size%202%20grps%20cohort.htm. The calculated minimal sample size needed for the study to be carried out was 1014 patients (statistical power set at 85%). The statistical analysis was performed by SPSS statistical package, version 19.0 (SPSS Inc., IBM Company, Chicago, IL, USA). The data were summarized by frequencies and percentages for categorical variables and by mean values and standard deviation for continuous ones. For comparison of categorical variables we used independent χ^2^-test. The normality of distribution of continuous data was assessed by Shapiro-Wilk test. Analysis of variance (ANOVA) was used for comparison of parametric data. Logistic regression analysis was used to disclose risk factors for AF and ischemic stroke. All results were considered to be statistically significant for *p*-values <0.05.

## 3. Results

**Figure 1:**
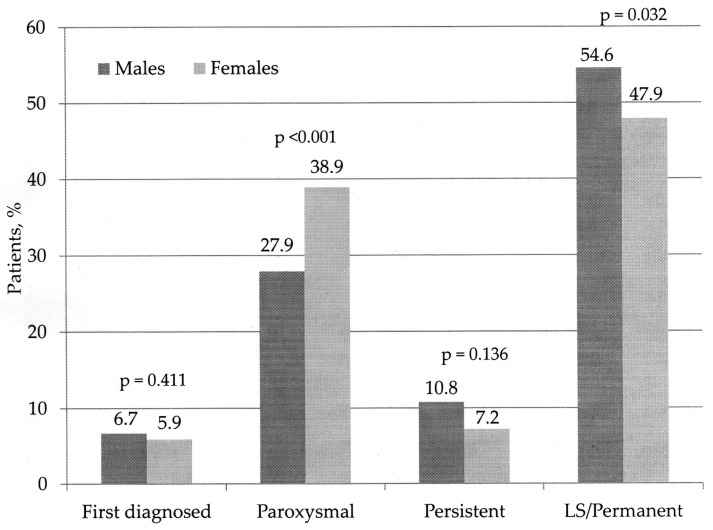
Gender differences in the types of AF; Independent χ^2^-test was used for comparison of the prevalence of different AF types in both genders; AF—atrial fibrillation; LS—long-standing persistent; *p*—level of significance.

Atrial fibrillation was diagnosed in 634 (61.7%) of all patients in our study, of them 315 males (49.7%). Figure 1 and Table 1 and Table 2 show the characteristics of the analyzed study population.

The mean heart rate of patients with first diagnosed AF at their disclosure (before application of rate control treatment) was 117.4 ± 14.6 beats per minute.

Heart failure was present in 627 (98.9%) patients with AF compared to 58 patients (14.8%) in sinus rhythm, *p* < 0.001. There was not significant gender difference in HF prevalence among AF patients: 311 males (49.6%) and 316 (50.4%) females, *p* = 0.627. In 74 (11.8%) of 627 AF patients with HF and 4 (6.9%) of 58 patients in sinus rhythm, HF was newly diagnosed after enrollment in our study, *p* < 0.001. Of all AF patients with HF, 40 (6.4%) had first diagnosed AF, 209 (33.3%)—paroxysmal, 55 (8.8%)—persistent and 323 (51.5%)—long-standing (LS)/permanent AF, *p* < 0.001. Third NYHA (New York Heart Association) class was most prevalent among AF patients—in 369 (58.9%), followed by II NYHA class—226 (36.0%), I NYHA class—21 (3.3%) and IV NYHA class—11 (1.8%), *p* < 0.001. There was not statistically significant gender difference regarding the NYHA class of AF population with HF.

The mean left ventricular ejection fraction (EF) of AF patients was 55.7 ± 11.4% compared to 59.6 ± 9.9% of patients in sinus rhythm, *p* = 0.116. The mean EF of patients with first diagnosed AF was 61.6 ± 8.6%, paroxysmal AF—59.9 ± 9.9%, persistent AF—57.6 ± 12.2%, LS/permanent AF—51.9 ± 11.7%, *p* < 0.001. Among the HF patients reduced left ventricular EF (HFrEF) <40% was found in 87 (13.9%), mid-range EF (HFmrEF) 40–49%—116 (18.5%) and HFpEF ≥50%—431 (68.7%), *p* < 0.001. There was not statistically significant difference by gender and type of AF regarding the sub-group analysis of EF groups. The association of different variables with AF is shown in Table 3.

The mean CHA_2_DS_2_-Vasc Score of AF patients, evaluating the risk for stroke and peripheral arterial embolic events was 3.83 ± 1.46 points (high risk): 3.43 ± 1.42 points for males and 4.13 ± 1.49 points for females, *p* = 0.023. The mean CHA_2_DS_2_-Vasc Score of patient with first diagnosed AF was 3.38 ± 1.40, paroxysmal AF—3.53 ± 1.53, persistent AF—3.32 ± 1.52, LS/permanent AF—4.08 ± 1.37 points, *p* = 0.003 for the last subgroup vs the others.

Among the 91 AF patients (14.4%) who had suffered ischemic stroke, 3 (3.3%) had first diagnosed AF, 24 (26.4%)-paroxysmal, 8 (8.8%)-persistent and 56 (61.5%)-LS/permanent AF, *p* < 0.001. The association of different variables with ischemic stroke is shown in Table 4.

The mean HAS-BLED Score of AF patients, evaluating the bleeding riskin anticoagulant treatment was 2.26 ± 0.97 points (moderate risk): 2.29 ± 1.01 points for males and 2.23 ± 0.93 points for females, *p* = 0.431. The mean HAS-BLED Score of patient with first diagnosed AF was 2.03 ± 0.80, paroxysmal AF—2.14 ± 1.02, persistent AF—2.16 ± 0.86, LS/permanent AF—2.39 ± 0.96 points, *p* = 0.224.

Oral antithrombotic treatment was prescribed to 543 (85.7%) of all AF patients: of them 265 (48.8%) males and 278 (51.2%) females, *p* = 0.382. Table 5 shows the prescription of different antithrombotics according to the type of AF.

The mean CHA2DS2-Vasc Score according to the oral antithrombotic treatment was: No treatment—3.6 ± 1.7 points, vitamin K antagonist (VKA)—3.9 ± 1.3 point, direct oral anticoagulants (DOACs)—3.3 ± 1.4, antiplatelets—4.0 ± 1.3 points; *p* no treatment/VKA = 0.009, *p* VKA/DOACs = 0.003, *p* DOACs/antiplatelet = 0.040; The mean HAS-BLED Score according to the oral antithrombotic treatment was: No treatment—2.2 ± 1.1 points, VKA—2.3 ± 0.9 points, DOACs—2.2 ± 0.8, antiplatelets—2.1 ± 1.0 points, *p* = 0.156.

Rate control treatment was administered to 478 (75.4%) AF patients: of them 232 (48.5%) males and 246 (51.5%) females, *p* = 0.332. Regarding the type of AF, rate control medication was administered to: first diagnosed—29 of 40 (72.5%), paroxysmal—133 of 212 (67.7%), persistent—37 of 57 (64.9%), LS/permanent—277 of 325 (85.2%), *p* < 0.001. Antiarrhythmic treatment was administered to 255 (40.2%): of them 128 (50.2%) males and 127 (49.8%) females, *p* = 0.512. Regarding the type of AF, antiarrhythmics were administered to: first diagnosed—22 of 40 (55.0%), paroxysmal—126 of 212 (59.4%), persistent—36 of 57 (63.2%), LS/permanent—71 of 325 (21.8%), *p* < 0.001. The drugs prescribed to our patients for rate and rhythm control are shown in Table 6.

## 4. Discussion

The purpose of our study was to characterize AF in a “real-life” population of in-hospital patients, irrespective of the reason for which they were admitted. We found high prevalence of AF—more than 60% of all patients had at least 1 episode of this rhythm disorder. Our results differed significantly from published data of other hospital-based studies in which AF ranged from 2% to 27% [2,3,4,10]. These discrepancies might be attributed to some extent to the inclusion criteria–much broader for our study and also to the risk profile of the patients: advanced age and high prevalence of concomitant RF/diseases. Our results are important because a large number of hospital physicians meet in their daily clinical practice patients similar to ours.

Generally, we did not find statistically significant gender difference in AF prevalence, except for the paroxysmal AF, more prevalent in females. According to other studies AF was less common in women [1,4,11,12,13,14]. The mean age of our AF population was significantly higher (69.6 years) compared to patients in sinus rhythm (64.4 years), a finding confirmed also by other authors [1,10,11,12,13]. Previous studies demonstrated that age >65 years was a major and independent non-modifiable RF for AF [1,2,3,4,5,6]. Our logistic regression analysis also disclosed age as an important RF for this dysrhythmia: age 65–74 years increased the risk for AF by ~1.4 and age ≥75 years-by ~1.6.

We found HF and HTN to be the most prevalent modifiable RF for AF, followed by VHD, chronic lung diseases and type 2 DM. Regarding HF, almost 2/3 of our AF patients had HFpEF and the percentage of HFrEF was significantly lower (~14%) compared to data from other clinical studies [2,3,4,5,6,7,8,13]. The surprisingly high prevalence of HFpEF in our study could be explained by the advanced age of the AF population and the high presence of co-morbidities such as HTN, VHD, type 2 DM, IHD. However, our logistic regression analysis showed that reduced EF exerted strongest impact on the risk for development of AF, followed by moderate to severe VHD. Arterial hypertension was stated by other authors as the most significant modifiable RF for AF [1,12,13,14,15,16,17,18,19]. Despite the high prevalence among our AF population, HTN was the third most influential RF for AF in our study according to the logistic regression analysis.

The prevalence of AF in patients with chronic lung diseases in our study was similar to data, reported by other authors [1,2,3,4,5]. Our logistic regression analysis did not disclose these diseases as factors exerting statistically significant influence on the risk for AF occurrence, but other authors reported that some chronic lung diseases such as obstructive sleep apnea and chronic obstructive pulmonary disease increased the adjusted hazard ratio for AF development by 1.5 to 2.44 [1,2,3,4,5,7].

The prevalence of type 2 DM among our AF population was significantly higher compared to other surveys [1,2,3,4,5,6,7]. However, our analysis showed it did not exert significant impact on AF occurrence. The published data we found about the independent role of type 2 DM for AF development were contradictory: according to some researchers type 2 DM was not a risk factor for AF at all and according to others type 2 DM was a very strong and independent AF risk factor [1,2,3,4,13].

Surprisingly, IHD was less prevalent than expected among our AF population. Although regression analysis showed that this disease increased the risk for AF occurrence by ~1.4, this effect was much weaker than anticipated. Other clinical studies found much higher IHD prevalence among AF patients and myocardial ischemia was acknowledged by many as the second most important modifiable RF for AF after HTN [1,2,3,4,5].

We found high cardioembolic risk for all types of AF in our study (CHA2DS2-Vasc >3 points) and ~14% of our AF patients had suffered an ischemic stroke. Reiffel et al. reported that AF accounted for >15% of all strokes in the USA, 36% of strokes in people >80 years of age, and ~20% of cryptogenic strokes [15]. Our regression analysis disclosed AF as the strongest risk factor for ischemic stroke of all variables we analyzed, followed by HTN and age ≥75 years. According to published data that we found, HTN was the strongest predictor for ischemic stroke, followed by cerebrovascular atherosclerotic disease, diabetes, smoking and advanced age. Non-anticoagulated atrial fibrillation was considered by most authors as the fourth or fifth most important RF for ischemic stroke [4,8,9,10,11].

Oral antithrombotic treatment was prescribed to 85.7% of our AF patients with VKAs slightly more preferred to DOACs (43.2% vs. 37.9% respectively). Possible explanations for such physician’s preference were the prevalence of VHD (i.e., valvular AF) in our study population and mostly the significantly higher cost of DOACs compared to VKAs in Bulgaria. The rate of oral anticoagulant treatment in AF patients, presented by other authors differs widely. Jain et al. reported that anticoagulant prescription in AF patients increased from 9% (1995–1998) to 30% (2011–2014) [20]. Weitz et al. found in their study that VKA prescription in AF decreased from 99% in 2010 to 67% by 2014 whereas DOACs administration increased to 33% [21]. The underuse of DOACs was not clinically justified even in terms of the bleeding risk. Large randomized clinical trials provided enough data proving the superiority of DOACs for embolic prevention and their more favorable bleeding-risk profile compared to VKAs [1,20,22].

Rate control treatment was prescribed to high percentage of our patients (~75%) with beta-blockers being the most preferred class. Digoxin had been applied only for acute heart rate control, when beta-blockers or verapamil did not have the necessary effect or in case of contraindications for their use. We tried to avoid chronic digoxin treatment because of reported data showing increased mortality in AF patients, treated continuously with this drug [23]. The clinical trials AFFIRM, RACE and HOT CAFÉ found that management of AF with the rhythm-control strategy offered no survival advantage over the rate-control strategy: the latter was associated with lower risk of adverse drug effects, reduced number of hospitalizations yearly and lower financial costs for patient’s treatment [1,22,23,24,25,26,27]. In the AFFIRM trial, beta-blockers, applied alone or in combination with digoxin achieved target heart rate in ~70% of the patients and calcium channel blockers only in 54% [1]. The results from the RATAF study differed significantly from those in the AFFIRM trial-calcium channel antagonists had superiority to other drugs regarding 24-h heart rate control, reduction of frequency and severity of symptoms [1,25].

Antiarhythmic treatment was administered to 40% of our patients with amiodarone being the most used antiarrhythmic drug. The possible explanation was the high prevalence of patients with HF and/or structural heart changes in which most of the representatives of class I and class III antiarrhythmics (Vaughan Williams classification) are contraindicated [1]. The PREFER registry including >7000 patients from 7 European countries showed that the three most commonly administered antiarrhythmic drugs in AF patients were amiodarone (51%), flecainide (22%) and sotalol (12%) [23].

Study limitations: 1. The number of patients we included in our single-center study does not allow us to formulate general conclusions about the entire AF population in Bulgaria; 2. We analyzed only hospitalized patients with AF and our results should not be extrapolated to out-hospital patients. The comorbidities in hospitalized patients might differ significantly from out-hospital patients thus influencing the incidence, prevalence and other characteristics of AF. 3. We analyzed some major but not all concomitant RF and diseases in patients with AF.

## 5. Conclusions

Atrial fibrillation was highly prevalent among our study population. Reduced and mid-range left ventricular ejection fraction, valvular heart disease and HTN were the risk factors with the strongest association with AF. Although a large number of our AF patients were administered antithrombotic treatment, the prescription rate of oral anticoagulants should be further improved.

## Figures and Tables

**Table 1:** Differences in age and prevalence of concomitant risk factors (RF) and diseases according to the presence and type of AF.

Risk Factors/Diseases	Sinus Rhythm *n* = 393	First Diagnosed AF *n* = 40	Paroxysmal AF *n* = 212	Persistent AF *n* = 57	LP/Permanent AF *n* = 325	*p*
Age, years	64.4 ± 11.0	67.2 ± 11.9	66.1 ± 12.8	68.4 ± 11.6	71.9 ± 8.6	<0.001
HTN, *n* (%)	381 (39.1)	38 (3.9)	200 (20.5)	56 (5.7)	299 (30.7)	0.394
IHD, *n* (%)	36 (33.6)	6 (5.6)	14 (13.1)	19 (17.8)	32 (29.9)	0.040
VHD *, *n* (%)	69 (21.0)	4 (1.2)	59 (18.5)	13 (4.0)	183 (55.8)	<0.001
Chronic lung disease, *n* (%)	116 (40.7)	13 (4.6)	70 (24.6)	18 (6.3)	68 (23.9)	<0.001
Type 2 DM, *n* (%)	119 (43.0)	7 (2.5)	45 (16.2)	12 (4.3)	94 (33.9)	0.112
Previous ischemic stroke, *n* (%)	32 (26.0)	3 (2.4)	24 (19.5)	8 (6.5)	56 (45.6)	0.014
Hypothyroidism, *n* (%)	41 (33.3)	5 (4.1)	39 (31.7)	8 (6.5)	30 (24.4)	0.068
Hyperthyroidism, *n* (%)	11 (31.4)	2 (5.7)	7 (20.0)	2 (5.7)	13 (37.1)	0.171

Age is presented as a mean value ± standard deviation; Independent χ^2^-test was used for comparison of categorical variables and ANOVA for parametric variables; * Moderate to severe insufficiency or/and stenosis; AF—atrial fibrillation; CVD—cerebro-vascular disease; DM—diabetes mellitus; HTN—arterial hypertension; IHD—ischemic heart disease; *p*—level of significance. The *p*-value refers to the comparison of sinus rhythm versus each category of atrial fibrillation; RF—risk factors; VHD—valvular heart disease.

**Table 2:** Gender differences in age and prevalence of concomitant RF and diseases in patients with AF.

Risk Factors/Diseases	Males *n* = 315	Females *n* = 319	*p*
Age, years	68.2 ± 9.7	70.5 ± 12.2	<0.001
Heart rate , beats per minute	84.7 ± 10.8	87.6 ± 11.5	0.267
HTN, *n* (%)	301 (50.8)	292 (49.2)	0.440
IHD, *n* (%)	48 (68.6)	23 (32.4)	0.002
VHD *, *n* (%)	110 (42.5)	149 (57.5)	0.003
-Mitral valve, *n* (%)	52 (41.9)	72 (58.1)	0.010
-Aortic valve, *n* (%)	18 (58.1)	13 (41.9)	0.010
-Mitral and aortic valve, *n* (%)	17 (42.5)	23 (57.5)	0.042
- Prosthetic valve, *n* (%)	23 (35.9)	41 (64.1)	0.023
Chronic lung disease, *n* (%)	95 (56.2)	74 (43.8)	0.020
Type 2 DM, *n* (%)	85 (53.8)	73 (46.2)	0.032
Previous ischemic stroke, *n* (%)	45 (49.5)	46 (50.5)	0.624
Hypothyroidism, *n* (%)	15 (18.3)	67 (81.7)	<0.001
Hyperthyroidism, *n* (%)	9 (37.5)	15 (62.5)	0.020

Age and heart rate are presented as a mean value ± standard deviation; Independent χ^2^-test was used for comparison of categorical variables and ANOVA for parametric variables; * Moderate to severe insufficiency or/and stenosis; AF—atrial fibrillation; DM—diabetes mellitus; HTN—arterial hypertension; IHD—ischemic heart disease; *p*—level of significance; RF—risk factors; VHD—valvular heart disease.

**Table 3:** Conditions/co-morbidities, associated with AF.

Variable	OR	95% Confidence Interval for OR		*p*
		Lower Limit	Upper Limit	
EF < 40%	1.951	1.208	3.151	<0.001
VHD	1.926	1.134	3.862	0.010
EF 40–49%	1.743	1.248	3.017	<0.001
HTN	1.653	1.092	3.458	<0.001
Age ≥ 75 years	1.625	1.019	2.915	0.032
Age 65–74 years	1.426	1.008	2.084	0.020
IHD	1.395	1.040	1.873	0.026

Univariate logistic regression was used for assessment of the independent influence of different variables on the risk for development of AF. Prior independent χ^2^-test was applied to identify categorical variables with statistically significant relationship to AF occurrence. These variables were entered into the univariate logistic regression model; AF—atrial fibrillation; EF—left ventricular ejection fraction; HTN—arterial hypertension; IHD—ischemic heart disease; OR—odds ratio for development of AF; *p*—level of significance; VHD—valvular heart disease.

**Table 4:** Conditions/co-morbidities, associated with ischemic stroke.

Variable	OR	95% Confidence Interval for OR		*p*
		Lower Limit	Upper Limit	
AF	1.793	1.151	2.792	<0.001
HTN	1.429	1.127	2.671	<0.001
Age ≥75 years	1.215	1.032	2.146	<0.001
Age 65–74 years	1.056	1.014	1.447	0.036

Univariate logistic regression was used for assessment of the independent influence of different variables on the risk of ischemic stroke. Prior independent χ^2^-test was applied to identify categorical variables with statistically significant relationship to stroke occurrence. These variables were further entered into the univariate logistic regression model; AF—atrial fibrillation; EF—left ventricular ejection fraction; HTN—arterial hypertension; IHD—ischemic heart disease; OR—odds ratio for development of AF; *p*—level of significance; VHD—valvular heart disease.

**Table 5:** Antithrombotic treatment according to the type of AF.

	AF *n* (%)				
	First Diagnosed *n* (%)	Paroxysmal *n* (%)	Persistent *n* (%)	LS/Permanent *n* (%)	*p*
**Treatment**					
No	20 (50.0)	33 (15.6)	14 (24.6)	24 (7.4)	<0.001
DOACs	5 (12.5)	54 (25.5)	15 (26.3)	166 (51.1)	0.044
VKA	13 (32.5)	119 (56.1)	21 (36.8)	121 (37.2)	0.221
Antiplatelets	2 (5.0)	6 (2.8)	7 (12.3)	14 (4.3)	0.354
Total	40 (100)	212 (100)	57 (100)	325 (100)	

Independent χ^2^-test was used for comparison of the presented categorical variables; AF—atrial fibrillation, LS—long-standing persistent, DOACs—direct oral anticoagulants (dabigatran, apixaban, rivaroxaban), *p*—level of significance; VKA—vitamin K antagonist.

**Table 6:** Medications, assigned to our patients for rate and rhythm control.

Medication	*n* (%)	*P*
**Rate control**		
Beta-blockers	379 (79.3)	
Calcium channel blockers	15 (3.1)	<0.0001
Digoxin *	84 (17.6)	
Total	478 (100)	
**Rhythm control**		
Amiodarone	111 (43.5)	
Propafenone	85 (33.3)	
Sotalol	47 (18.4)	0.03
Flecainide	12 (4.7)	
Total	255 (100)	

Independent χ^2^-test was used for comparison of the presented categorical variables; *p*—level of significance; * Used only for acute heart rate control.
